# Novel side chain functionalized polystyrene/O-PBI blends with high alkaline stability for anion exchange membrane water electrolysis (AEMWE)†

**DOI:** 10.1039/d3ta02978f

**Published:** 2023-10-11

**Authors:** Linus Hager, Manuel Hegelheimer, Julian Stonawski, Anna T. S. Freiberg, Camilo Jaramillo-Hernández, Gonzalo Abellán, Andreas Hutzler, Thomas Böhm, Simon Thiele, Jochen Kerres

**Affiliations:** a Forschungszentrum Jülich GmbH, Helmholtz Institute Erlangen-Nürnberg for Renewable Energy (IEK-11) Cauerstr. 1 91058 Erlangen Germany l.hager@fz-juelich.de; b Department of Chemical and Biological Engineering, Friedrich Alexander Universität Erlangen-Nürnberg Egerlandstr. 3 91058 Erlangen Germany; c Institute of Molecular Science, University of Valencia c/ Catedrático José Beltrán 2 Paterna Spain; d Chemical Resource Beneficiation Faculty of Natural Sciences, North-West University Potchefstroom 2520 South Africa

## Abstract

We report the synthesis of a polystyrene-based anion exchange polymer bearing the cationic charge at a C6-spacer. The polymer is prepared by a functionalized monomer strategy. First, a copper halide catalyzed C–C coupling reaction between a styryl Grignard and 1,6-dibromohexane is applied, followed by quaternization with *N*-methylpiperidine and free radical polymerization. The novel polymer is blended with the polybenzimidazole O-PBI to yield mechanically stable blend membranes representing a new class of anion exchange membranes. In this regard, the ratio of the novel anion exchange polymer to O-PBI is varied to study the influence on water uptake and ionic conductivity. Blend membranes with IECs between 1.58 meq. OH^−^ g^−1^ and 2.20 meq. OH^−^ g^−1^ are prepared. The latter shows excellent performance in AEMWE, reaching 2.0 A cm^−2^ below 1.8 V in 1 M KOH at 70 °C, with a minor degradation rate from the start. The blend membranes show no conductivity loss after immersion in 1 M KOH at 85 °C for six weeks indicating high alkaline stability.

## Abstract 2

A new material comprising a functionalized polystyrene and O-PBI showing excellent stability and performance in anion exchange membrane water electrolysis is introduced.

## Graphical abstract

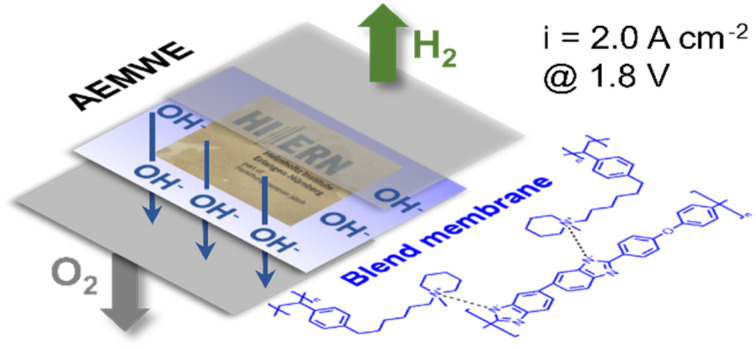

## Introduction

Long-term energy storage is crucial for the transition from fossil fuels to renewable energies due to the fluctuating energy generation of renewable energy sources such as solar power or wind energy.^1,2^ Water electrolysis could produce hydrogen from water and electricity from renewable energy resources.^3^ Consequently, hydrogen, generated from solar, wind, or other renewable energy technologies, could serve as a carbon-neutral energy carrier in many essential areas, such as the chemical industry, steel production, and vehicle fuel.^3^

Today, the two mature electrolysis technologies used for hydrogen production are alkaline water electrolysis (AWE) and proton exchange membrane water electrolysis (PEMWE). However, both technologies suffer from specific disadvantages, but on the other hand, they also offer certain benefits.

AWE is typically operated with 5.5 to 8.0 M KOH as an electrolyte. A porous diaphragm separating the anode and cathode enables electrolyte transport but hinders the mixture of the produced hydrogen and oxygen.^4,5^ Under alkaline conditions, non-noble electrocatalysts could be used for the oxygen evolution reaction (OER) and the hydrogen evolution reaction (HER) in comparison to PEMWE, where typically only platinum group metals (PGMs) are stable.^6–10^ However, using a diaphragm requires comparatively large distances between the anode and cathode (<2 mm) to prevent a mixture of H_2_ and O_2_. Thus, the maximum current density in AWE is limited to approximately 0.25 A cm^−2^ due to the dependency of the ionic resistance on the electrolyte thickness.^3,11–13^

In PEMWE, a solid polymer electrolyte, conductive for protons, directly contacts the electrodes. This has the advantage that due to the decreased electrolyte thickness (50 to 200 μm), the ionic resistance is reduced. Consequently, these so-called “zero-gap” electrolyzers could achieve higher current densities. This ultimately leads to higher hydrogen production rates compared to AWE.^14^ Furthermore, due to the solid electrolyte, the gas crossover is decreased, and the operation with different pressures at the anode and cathode is possible.^15^ With PEMWE, current densities are achieved in the range of 1 to 3 A cm^−2^ and lifetimes of 15 000 to 20 000 h.^3^ The main disadvantage of PEMWE is the necessity to use PGMs as electrocatalysts and other highly corrosion-resistant materials, which typically results in higher investment costs.^8^

Recent research aims at a combination of the advantages of AWE and the zero-gap architecture of PEMWE. Anion exchange membrane water electrolysis (AEMWE) operates at high pH to enable the use of non-noble electrocatalysts. In addition, a hydroxide conductive membrane is used as a solid electrolyte instead of a diaphragm.^16^ Typically, in AEMWE, 1 M KOH is used as feed. Limitations of AEMWE are the lower hydroxide conductivity compared to proton conductivity and, consequently, lower current densities and lower hydrogen production rates. Furthermore, the chemical stability of AEMs in alkaline media still limits the technical applicability of this technology.^16–18^

In recent years, the performance of AEMWE increased significantly since current densities higher than 2.0 A cm^−2^ at voltages below 2.0 V were already achieved with reduced amounts of PGM electrocatalysts.^2,19–26^ Finally, electrolysis with pure water also was demonstrated with polyfluorenes bearing the quaternary ammonium groups in long side chains.^27^

Furthermore, ion-solvating membranes have recently been used as an intermediate between AWE and AEMWE.^28^ Contrary to AEMs, ion-solvating membranes work with concentrated KOH (typically 24 wt%) by using membranes that swell in the presence of KOH. Compared to the diaphragms used in AWE, ion-solvating membranes are dense and thus allow to be used in the zero-gap architecture. However, compared to AEMWE, concentrated KOH is necessary for sufficient conductivity of the membranes, which can cause damage to ion-solvating materials like poly(2,20-(*m*-phenylene)-5,5′-bibenzimidazole) (*m*-PBI).^28^ Interestingly, with *m*-PBI as an ion-solvating membrane and 24 wt% KOH as an electrolyte, an electrolyzer achieved 1.7 A cm^−2^ at 1.8 V, reaching the performance of AEMWE.^28^ Using *m*-PBI as an ion-solvating membrane with 24 wt% KOH led to significant membrane degradation and, finally, membrane failure due to hole formation after 309 h.^28^

One commercially available membrane applied in AEMWE is Sustainion®. Sustainion® is a 4-vinylbenzyl chloride styrene copolymer quaternized with 1,2,4,5-tetramethylimidazole.^18,29^ Sustainion® shows excellent performance in AEMWE and CO_2_ electrolysis.^25,30–36^ However, on other polymer systems, it was found that the conductivity and alkaline stability could be increased if the cationic group is separated from the backbone by an alkyl spacer, which was attributed to phase separation effects resulting in conductive ion channels.^37–40^ Consequently, in this study, we aimed to synthesize a side-chain functionalized polystyrene bearing the cationic group at the end of a C6-alkyl spacer. In 1996, Tomoi *et al.* reported the synthesis of anion exchange resins based on styrene monomers with alkyl bromides in the *para* position.^41^ The quaternized and crosslinked resins with alkyl spacers were superior regarding alkaline stability compared to their benzyl trimethylammonium analogs.^41^ More recently, Ponce-González *et al.* grafted styrene monomers with the cationic group at the end of a C4 spacer on radiated ETFE foils, which had exceptional alkaline stabilities combined with high performance in anion exchange membrane fuel cells.^42^ Ertem and Coughlin recently compared the alkaline stability of trimethylammonium cations attached to a phenyl unit by a hexyl spacer to the C1-spaced cation. With ^1^H-NMR spectroscopy, they proved that for the hexyl-spaced TMA cations, less degradation occurred compared to the ammonium group in the benzylic position.^40^

In addition, to enhance the stability of polystyrene-based AEMs, this study focused on circumventing the brittleness of polystyrenes by a blending approach exploiting the excellent mechanical properties of polybenzimidazoles.^18^ Previous studies showed that polybenzimidazoles could be deprotonated under alkaline conditions, whereby the negatively charged benzimidazole units could form ionic crosslinks with the positively charged anion exchange polymers.^43^ This approach could render water-soluble polymers water-insoluble, and an initially mechanically brittle material could be embedded in a mechanically stable matrix. Compared to pure PBI, which is non-conductive in 1 M KOH, a blend approach with an anion-conducting polymer could lead to membranes with intrinsic hydroxide conductivity, allowing their use in applications like AEMWE with 1 M KOH, which also decreases the probability of membrane degradation.

Therefore, this study presents the synthesis of 4-(6-bromohexyl)styrene followed by quaternization of the bromine group with *N*-methylpiperidine. The fully quaternized 1-methyl-(6-(4-vinylphenyl)hexyl)piperidin-1-ium bromide is subsequently polymerized *via* free radical polymerization to yield a distinctly novel anion exchange polymer (P4HexPipSt). After blending with the polybenzimidazole poly[2,2′-(*p*-oxydiphenylene)-5,5′-bibenzimidazole] (O-PBI), the membranes are analyzed regarding conductivity, water uptake, and alkaline stability. Finally, their performance in AEMWE is investigated, whereby the non-PGM catalyst Ni–Fe LDH is used at the anode side. The actual cell performance is compared to Aemion+® as a commercial reference. To the best of our knowledge, this study presents for the first time the successful application of a blend membrane in AEMWE with better performance than a commercially available AEM like Aemion+®. This demonstrates the potential of this material design concept for tailoring the material properties for further improvement toward novel high-performance anion exchange membranes.

## Experimental

### Materials

#### Free radical polymerization of 1-methyl-(6-(4-vinylphenyl)hexyl)piperidin-1-ium bromide

1-Methyl-(6-(4-vinylphenyl)hexyl)piperidin-1-ium bromide (8.372 g, 22.851 mmol, 1.00 eq.) was dissolved in a 50 : 50 vol% DMF/H_2_O mixture (11.40 mL). Afterward, the reaction mixture was degassed by bubbling argon through the solution for 30 min. After adding AIBN (15.50 mg, 97.40 μmol, 0.004 eq.) as initiator, the reaction mixture was stirred at 65 °C for 24 h. The polymer P4HexPipSt was purified by dialysis against ultrapure water for 7 d and obtained after lyophilization. Yield: 7.850 g (94%). ^1^H NMR (500 MHz, DMSO-*d*_6_, *δ*): 6.90–6.39 (m, 4H, Ar *H*), 3.43–3.40 (m, 6H, C*H*_2_–N^+^), 3.08 (s, 3H, C*H*_3_–N^+^), 2.46 (m, 3H, C*H*_2_–Ar and C*H*–Ar), 1.80–1.30 (m, 16H, C*H*_2_).

#### Blend membrane preparation

Poly(1-methyl-(6-(4-vinylphenyl)hexyl)piperidin-1-ium bromide) (P4HexPipSt, 1.000 g) was dissolved in DMSO (12.521 g) by stirring at 80 °C for 1 d. Afterward, a 5 wt% O-PBI solution in DMSO (6.178 g) was added, and the polymer mixture was homogenized by gentle stirring at 80 °C for 1 d. The polymer solution was cast on a glass slide and doctor-bladed (gap height: 1.000 mm). After evaporating the solvent at 110 °C for 2 h, the membrane was detached from the glass slide by immersing it in ultrapure water. The membrane was purified by immersion in 1 M HCl for 24 h at 85 °C, followed by immersion in 1 M NaCl for 24 h at 85 °C two times, and finally by immersion in H_2_O at 85 °C for 24 h. The membrane was dried under vacuum at 60 °C. By this procedure, membranes with a thickness of 50 μm were obtained. The membrane obtained by this procedure had an IEC of 2.20 mmol g^−1^. By varying the ratio of P4HexPipSt to O-PBI blend membranes with different IECs could be obtained.

### Methods

#### Membrane testing in anion exchange membrane water electrolysis (AEMWE)

The membrane electrode assembly (MEA) was tested in an AEMWE cell fixture for single-cell tests with an active area of 5 cm^2^. The bipolar plates out of monel had a single-channel serpentine pattern with a channel width of 1 mm and a land width of 0.8 mm. A thin layer of gold was applied to the bipolar plates to prevent the passivation of the material. Matteco LDH catalyst (1st gen.) NiFe-LDH, provided by Matteco, was used as an anode catalyst with 10 wt% Aemion® (AP1-HNN8-00) as a binder. The catalyst ink solution (1% solid content, solvents: ethanol : water 1 : 1) was spray coated on a sintered nickel fiber substrate (Bekaert Bekipor 2NI 18-0,25) using a Sono-Tek Exactacoat device. For the cathode, platinum on carbon (platinum nominally 60% on high surface area advanced carbon support from ThermoFisher) was spray coated on a carbon gas diffusion layer (Freudenberg H24C5) with 10 wt% Aemion® ionomer (AP1-HNN8-00). The resulting loading was 2.0 mg cm^−2^ for the anode and 0.5 mg cm^−2^ Pt for the cathode. Membrane samples of roughly 5 cm × 5 cm were used for the test. The P4HexPipSt/O-PBI blend membranes were preconditioned in 1 M KOH for 24 h, the commercially available Aemion+® membranes (AF3-HWK9-75-X 75) in 1 M NaCl for 24 h, and 1 M KOH for 36 h, as instructed by Ionomr. For MEA preparation, the catalyst-coated electrodes were placed in glass-fiber reinforced PTFE frames, with the membrane clamped between them. Twelve M8 screws were tightened to a torque of 10 Nm to seal the cell assembly. PTFE frames with a thickness of 235 μm each were used for the 270 μm nickel PTL and 230 μm carbon GDL aiming for an average pressure of 2.5 to 3 MPa on the active membrane area. Testing was conducted at 70 °C and atmospheric pressure on both sides. The feed solution was 1 M KOH with a constant flow rate of 30 mL min^−1^. The testing procedure consisted of the following main steps:

1. Test for electrical short.

2. Cell break-in at 1.8 V for 1 h.

3. Polarization curve (0.02–4 A cm^−2^ with 3 min holding time; impedance scans: 150 kHz to 1 Hz).

4. Constant current at 1 A cm^−2^ for 15 h.

5. Second polarization curve.

## Results and discussion

### Monomer and polymer synthesis

**Fig. 1:**
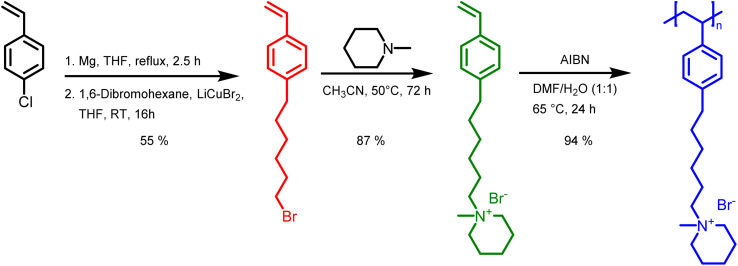
Synthesis of poly(1-methyl-(6-(4-vinylphenyl)hexyl)piperidin-1-ium bromide): (1) coupling of 1,6-dibromohexane to a styryl Grignard. (2) Quaternization with *N*-methylpiperidine. (3) Free radical polymerization to yield P4HexPipSt.

**Fig. 2:**
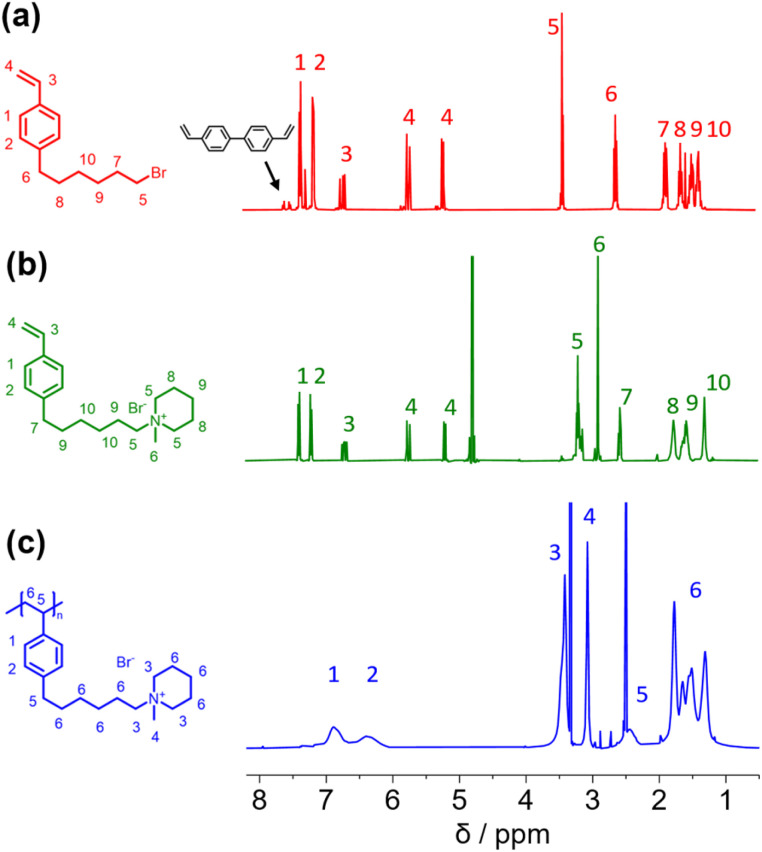
(a) ^1^H NMR spectra of 4-(6-bromohexyl)styrene (b) 1-methyl-(6-(4-vinylphenyl)hexyl)piperidin-1-ium bromide (c) poly(1-methyl-(6-(4-vinylphenyl)hexyl)piperidin-1-ium bromide).

In this study, we report the synthesis of a novel anion exchange polymer based on a polystyrene backbone with an alkyl spacer (C6) between the backbone and the quaternary ammonium group (Fig. 1). This polymer was blended with O-PBI to obtain highly stable membranes suitable for anion exchange membrane water electrolysis. We obtained 4-(6-bromohexyl)styrene by coupling the Grignard reagent from 4-chlorostyrene to a significant excess of 1,6-dibromohexane with a yield of 55% (Fig. 1). The ^1^H-NMR spectrum of the target compound (Fig. 2a) shows all relevant signals corresponding to the protons in 4-(6-bromohexyl)styrene. Additional peaks are observed in the ^1^H-NMR spectrum next to the aromatic and vinylic protons of the target compound (Fig. 2a). Most likely, these signals correspond to 4,4′-divinyl-1,1′-biphenyl, formed as a side product by coupling two styryl Grignard reagents catalysed by the copper halide. It is crucial to remove 4,4′-divinyl-1,1′-biphenyl, because it could act as a crosslinker during free radical polymerization leading to insoluble materials. We assumed that the crosslinker could be removed after conversion of the bromine substituent in a quaternary ammonium group due to different solubilities in organic solvents. To obtain the positively charged monomer, a Menschutkin reaction of 4-(6-bromohexyl)styrene with *N*-methylpiperidine was performed (Fig. 1), whereby the monomer 1-methyl-(6-(4-vinylphenyl)hexyl)piperidin-1-ium bromide was obtained with a yield of 87% (Fig. 1).

After mixing with diethyl ether and subsequent filtration, we received the quaternary ammonium functionalized monomer with an increased purity resulting from the different solubility properties of 4,4′-divinyl-1,1′-biphenyl and the quaternized monomer (Fig. 2b). In the ^13^C NMR spectrum, all signals can be assigned to the target compound (Fig. S3†).

In the next step, we aimed to polymerize the new monomer to obtain the C6-spaced quaternary ammonium functionalized polystyrene (Fig. 1), whereby standard conditions for free radical polymerization, according to Hemp *et al.*, were applied.^44^ The pure polymer was obtained with a high yield after dialysis against ultrapure water and lyophilization. The ^1^H NMR spectrum indicates the formation of the polymer since all integrals found match perfectly the expected molecular structure of the polymer (Fig. 2c). The positively charged polymer exhibits excellent solubility in polar solvents like methanol, ethanol, and dimethyl sulfoxide. Moreover, it is also soluble in H_2_O, which limits its applicability as pure material in aqueous electrochemical energy applications such as AEMWE. Consequently, in this study, we focused on blends of this polymer with the polybenzimidazole O-PBI. Before using the newly synthesized polymer as an anion-conducting part in a blend with O-PBI, the basic polymer properties were investigated. The polymer (Fig. S4a†) was analyzed regarding its thermal stability utilizing thermogravimetry (Fig. S4b†). It was found that the polymer shows the same high thermal stability under an oxygen-containing atmosphere and nitrogen. The first degradation step starts at 273 °C under nitrogen and 271 °C under synthetic air. Furthermore, typically for ion-containing polymers, the first 50 wt% weight loss corresponds to the cationic group's decomposition.^45^ The thermal stability of the polymer is high enough to use it as an ion-conducting component in AEMWE at 70 °C. Next, we investigated the side-chain functionalized polystyrene ionomer's thermal transitions *via* differential scanning calorimetry (Fig. S4c†). Interestingly, the polymer shows a glass transition below its decomposition temperature. The glass transition temperature was determined to be 103 °C, which is almost identical to the glass transitions of pure polystyrene without any functional group, typically reported as 100 °C.^46^ The glass transition of 103 °C results from two contrary effects on chain mobility. On the one hand, alkyl chains in *para*-position are known to significantly decrease the glass transition temperature of polystyrene.^47^ On the other hand, ionic functional groups increase the glass transition due to the ionic interactions limiting chain mobility. The novel polymer's molecular weight was analyzed using gel permeation chromatography with DMSO as eluent and narrowly distributed PMMA standards as calibration (Fig. S4d†). A molecular weight of 30 kg mol^−1^ with a dispersity of 1.28 was obtained, indicating the successful polymerization of the self-synthesized monomer. To summarize, a novel side-chain functionalized styrene monomer was synthesized and subsequently polymerized by free radical polymerization to the functionalized polymeric quaternary ammonium-containing polymer.

### Blends of P4HexPipSt with the polybenzimidazole O-PBI

It is well accepted that anion exchange membranes based on polymer blends with polybenzimidazoles could exhibit good to excellent alkaline stabilities.^48–52^ Water-soluble polymers could be water-insoluble by blending with water-insoluble polymers *via* chain entanglement between both polymer chains.^43,53–55^ However, to the best of our knowledge, none of these blend membranes were used in AEMWE at industrially relevant current densities or in comparison to state-of-the-art materials.

**Fig. 3:**
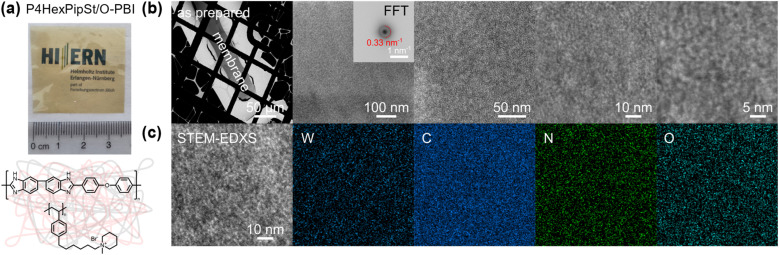
(a) Image of the blend membrane with a P4HexPipSt content of 73 wt% and structure of the blend components. (b) HAADF-STEM images of the as prepared membrane. (c) STEM-EDX spectrum images showcasing the initial homogeneous distribution of C, N, and O, as well as W utilized for staining. Structure size within the membrane was evaluated *via* the radial profile of the fast Fourier transformed micrograph (FFT) shown in (b).

The polybenzimidazole O-PBI was used due to its excellent mechanical properties, its solubility in DMSO, and the hydrophobicity necessary to achieve water-insoluble polymer blends. Furthermore, we were motivated to combine the reported excellent alkaline stability of side-functionalized polystyrenes with the mechanical robustness of O-PBI to prepare distinctly novel AEMs since a blend of side-chain functionalized polystyrene with a polybenzimidazole has not been reported so far. Our blend experiments were guided by calculations of the theoretical ion exchange capacities (IEC) since these values represent the number of charges in the membranes. We tried different weight ratios of O-PBI and poly(1-methyl-(6-(4-vinylphenyl)hexyl)piperidin-1-ium bromide), whereby theoretical IECs between 2.0 mmol g^−1^ and 2.4 mmol g^−1^ were targeted. The calculation of theoretical IEC values is given in the ESI.† The corresponding titrated IECs were lower, between 1.58 mmol g^−1^ and 2.20 mmol g^−1^. Fig. S5† shows the titrated IECs dependent on the targeted theoretical values and the corresponding amount of P4HexPipSt in the blend. The lower titrated IECs probably result from an effect of the O-PBI component, which could cause an additional impact on the counterions. However, by increasing the P4HexPipSt content in the blend, we observed an increase in the titrated IECs (Fig. S5†). By exploiting the superior solubility of the novel cationic polymer in DMSO, we prepared completely homogeneous and robust blend membranes with O-PBI (Fig. 3a). To check whether the P4HexPipSt is permanently fixed in the blend, we measured the dry mass of different membrane samples with varying IECs after immersion in H_2_O for different time intervals. We compared it to the initial mass by calculating the relative mass change (Fig. S6†). Interestingly, the membranes show slightly higher masses after immersion in H_2_O, even after drying under a vacuum at 120 °C for 24 h. Thus, within the accuracy of the balance, we did not observe the dissolution of P4HexPipSt from the blend membrane, indicating the high strength of the physical entanglement of the polymer chains.

**Fig. 4:**
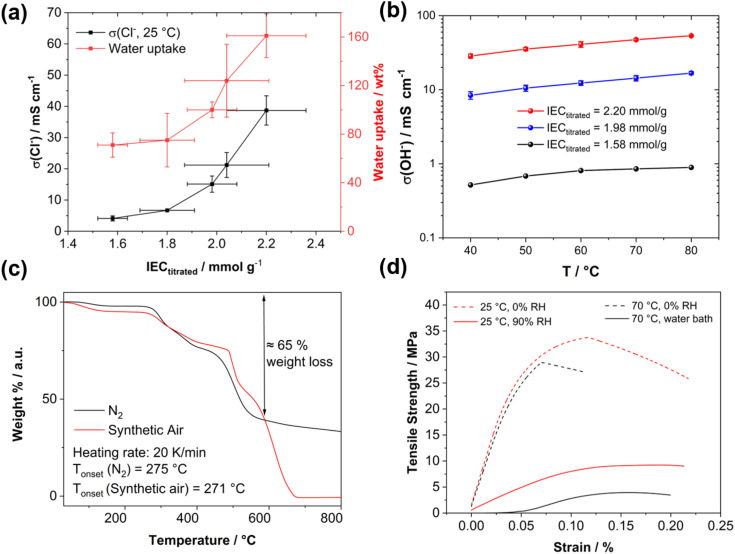
(a) Chloride conductivity at room temperature and water uptake at 85 °C dependent on the titrated IEC. (b) Temperature-dependent conductivity of a blend membrane with a titrated IEC of 2.20 mmol g^−1^ under 95% relative humidity in its OH^−^ form. (c) TGA curves of a blend membrane with a P4HexPipSt content of 65 wt% corresponding to an IEC of 1.58 mmol g^−1^. (d) Stress–strain curves of an exemplary blend membrane with an IEC of 2.20 mmol g^−1^ under different degrees of hydration (0% RH, 90% RH, fully hydrated) and different temperatures (25 °C and 70 °C).

High-angle annular dark field (HAADF) scanning transmission electron microscopy (STEM) imaging of an ultra-thin cross-section was performed to demonstrate the uniformity of the blend membrane (Fig. 3b). To gain a mass-thickness contrast, the counter ion in the membranes was exchanged to WO_4_^2−^. The WO_4_^2−^ anions are predominantly accumulated at the positively charged ammonium groups of P4HexPipSt. Since more electrons are scattered toward higher angles at the WO_4_^2−^ anions (*Z* contrast), the regions with an increased amount of WO_4_^2−^ appear as bright areas in the HAADF-STEM image. A homogeneous microstructure is observed since no large phase-separated areas appear, and uniform distribution of the WO_4_^2−^ rich spots over the investigated region indicates that P4HexPipSt and O-PBI formed a homogeneous blend membrane on the molecular level. The structure size was analyzed *via* fitting of the background-corrected, polar-integrated radial profile of the fast Fourier transformed micrograph (FFT),^56^ whereby an average structure size of 3.0 nm was determined. This structure size originates from the introduction of the alkyl spacer between the hydrophobic backbone and the hydrophilic piperidinium group, whereby the TEM image obtained for our blend system is consistent with the microstructure of other polymer systems comprising hydrophobic backbones and cationic groups separated from the backbone by long alkyl spacers.^57^ Additional energy dispersive X-ray (EDX) spectrum imaging (Fig. 3c) was used to analyze the distribution of the different elements in the blend membrane. The spatial distribution of tungsten and nitrogen corresponds well, indicating that the WO_4_^2−^ anion is mainly accumulated at the nitrogen-containing ammonium groups. However, also the O-PBI component contains nitrogen, which can explain slight deviations in the spatial distribution of tungsten and nitrogen. Again, both elements are uniformly distributed. Since carbon is the most abundant element in both polymers, the STEM-EDX spectrum image shows a high intensity in all regions. Because the WO_4_^2−^ anions and O-PBI contain oxygen, the oxygen signal appears in the bright W-rich hydrophilic and darker parts, mainly corresponding to O-PBI. Through-plane conductivities of the blend membranes were analyzed by electrochemical impedance spectroscopy using aqueous 1 M NaCl as an electrolyte. The results are shown in Fig. 4a. With increasing the P4HexPipSt content in the blend, the conductivity increases non-linearly. For low IECs until 1.98 mmol g^−1^, only a slow conductivity increase was observed. From this point, a sharp rise in conductivity occurs. Simultaneously, the water uptake at 85 °C increases to 160 wt% for the membrane with the highest P4HexPipSt content. We assume that the percolation threshold is reached at this point, which has already been shown for other polymer systems.^58^ It should be mentioned that the water uptake was measured at 85 °C with pure water. Under these conditions, AEMs typically take up more water than at room temperature, which already was shown with other membranes.^45,59^ Consequently, adjusting the ratio of both blend components can cover various conductivities. Furthermore, the swelling ratio in the longitudinal direction (SR_L_) and the transversal direction (SR_T_) follow the same trend as the water uptake (Fig. S7†). To investigate the conductivity of the membranes without applying an external electrolyte, the in-plane conductivity of the membranes in their hydroxide form was measured *via* the four-point probe method under 95% RH (Fig. 4b). The conductivity for a blend with a titrated IEC of 2.20 mmol g^−1^ increases from 30 mS cm^−1^ at 40 °C to 54 mS cm^−1^ at 80 °C. Interestingly, when comparing blends with different IECs, the conductivity drops drastically when the IEC decreases from 2.20 mmol g^−1^ to 1.98 mmol g^−1^ (Fig. 4b). By decreasing the IEC even further to 1.58 mmol g^−1^, the membrane loses almost all its conductivity. The conductivity obtained under relative humidity without an external electrolyte correlates well with the Cl^−^ conductivity obtained with 1 M NaCl as an electrolyte. Here, we also observed a sharp increase in conductivity when the IEC was increased from 1.98 mmol g^−1^ to 2.20 mmol g^−1^.

Exemplary, a blend membrane with a P4HexPipSt content of 61 wt% corresponding to an IEC of 1.58 mmol g^−1^ was analyzed with TGA to verify the P4HexPipSt content in the blend and the thermal stability (Fig. 4c). In both cases, the thermal stability under synthetic air and nitrogen is excellent until around 270 °C. Degradation starts at this temperature, whereby until about 600 °C, most of the P4HexPipSt is degraded. The remaining mass mainly corresponds to O-PBI, which only wholly degrades under synthetic air conditions.

Finally, the prepared blend membranes' mechanical properties were investigated using dynamical mechanical analysis in tensile mode (DMA, Fig. 4d). An exemplary membrane with a P4HexPipSt content of 73 wt% corresponding to an IEC of 2.20 mmol g^−1^ (Fig. S5†) was analyzed at different temperatures and humidities. Interestingly, the membrane behaves similarly under dry conditions (0% RH) at different temperatures (25 °C and 70 °C). The Young's modulus decreases slightly from 617 MPa at 25 °C to 564 MPa at 70 °C. After surpassing a maximum, the membranes show a creeping behavior, which on a molecular scale, corresponds to the de-entanglement of the polymer chains in the blend.

Moreover, the membrane was measured at 25 °C and 90% RH to investigate the influence of water uptake on the membrane's mechanical properties. The Young's modulus decreased significantly to 87 MPa, indicating a softening effect due to water uptake. Measuring the membranes under similar conditions as in the application (70 °C, fully immersed in H_2_O) is desirable to get information about the mechanical properties under actual working conditions. Consequently, the blend membrane was immersed in a water bath, and the stress–strain curve was measured again. The Young's modulus decreases to 53 MPa, indicating more water uptake than the measurement at 25 °C. Furthermore, creeping behavior is also reduced. Summarizing the DMA analysis, a sufficiently high anion conductivity is reached at a P4HexPipSt content of 73 wt% corresponding to an IEC of 2.20 mmol g^−1^, whereas the DMA results show that the O-PBI still can dominate the mechanical properties, which also was proven by DMA analysis of pure O-PBI (Fig. S9†).

**Fig. 5:**
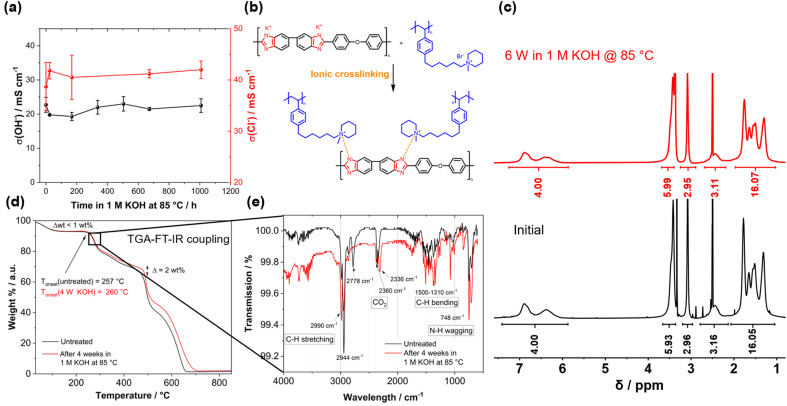
(a) Conductivity after treating a P4HexPipSt/O-PBI membrane with an IEC of 2.20 mmol g^−1^ with 1 M KOH at 85 °C for different time intervals. (b) Ionic crosslinks formed after treating the blend membranes with KOH. (c) ^1^H NMR analysis of P4HexPipSt dissolved in 1 M KOH at 85 °C for 6 weeks compared to the untreated polymer. (d) TGA measurement of a blend membrane with an IEC of 2.20 mmol g^−1^ before and after treatment with 1 M KOH at 85 °C for 4 weeks. (e) IR spectrum of the gaseous degradation products of the first degradation step.

Before testing the novel blend membranes in AEMWE, the alkaline stability was investigated *ex situ* (Fig. 5a). For this purpose, a P4HexPipSt/O-PBI membrane with an IEC of 2.20 mmol g^−1^ was immersed in 1 M KOH at 85 °C for different intervals. This membrane was chosen due to its promising anion conductivity and sufficient mechanical stability. Before analyzing the membranes, a counterion exchange back to Cl^−^ was performed to compare the membranes to the pristine samples. Counterion exchange was achieved by immersing the blend membranes in 1 M HCl at 85 °C for 24 h, followed by immersion in 1 M NaCl and DI water each for 24 h at 85 °C. We did not observe a decrease in the Cl^−^ conductivity (Fig. 5a), indicating high chemical stability of the P4HexPipSt anion exchange polymer. The OH^−^ conductivity of the aged samples was also measured under 95% RH without an external electrolyte to eliminate the influence of the ion-conducting 1 M NaCl electrolyte on the measurement. The results are compared to the measurement with 1 M NaCl in Fig. 5a. The conductivity under 95% RH is lower than the values obtained with 1 M NaCl as an electrolyte due to the absence of a conducting electrolyte and relative humidity instead of complete hydration (compare Fig. 4). However, we did not observe a drop in OH^−^ conductivity after immersion in 1 M KOH at 85 °C for 1000 h, indicating high stability of the blend membrane under the investigated conditions. Interestingly, the membranes were insoluble before and after the counterion exchange, showing a high strength of chain entanglement in the blend. Moreover, ionic crosslinks could be formed during the stress test in KOH (Fig. 5b), which are not fully broken during the counter ion exchange. These ionic crosslinks could additionally mechanically stabilize the blend membranes in an alkaline environment. Thus, we could not dissolve the alkaline-treated membranes for analysis *via* NMR spectroscopy. However, to investigate whether the piperidinium group degraded during the KOH treatment, we dissolved pure P4HexPipSt in 1 M KOH and heated the solution to 85 °C for 6 weeks. Finally, the ^1^H NMR spectra of the pristine and aged polymer were compared (Fig. 5c). No changes in the ^1^H NMR spectrum after the KOH treatment were detected, and all integrals stayed unchanged. Thus, we are confident that the piperidinium group has not degraded within the investigated time interval.

In addition to the conductivity investigation and the NMR analysis of P4HexPipSt, we focused on TGA curves. The latter was already applied in analyzing the degradation behavior of anion exchange membranes since it effectively detects differences in cationic group loss (Fig. 5c).^60–62^ The TGA curves of the membranes before and after KOH treatment are almost identical until 400 °C. Both show a clear degradation step at 260 °C, corresponding to the loss of the piperidinium group of P4HexPipSt (Fig. S4†). This was further verified by utilizing FT IR spectroscopy of the gaseous degradation products of the first degradation step (Fig. 5e). The typical C–H stretching vibration bands in the FT-IR spectrum are observed at 2990 cm^−1^. In the fingerprint region, the C–H bending vibrations are observed at 1500–1310 cm^−1^. Interestingly, at 748 cm^−1^, a vibration band is observed, which could be assigned to the N–H wagging vibration. Thus, it could be confirmed that at the first thermal degradation step, the *N*-methylpiperidinium group is lost. At 2360 cm^−1^ and 2336 cm^−1^, a vibration band typical for CO_2_ is observed, which results from air in the IR measurement cell and not from the sample. Furthermore, from the TGA traces shown in Fig. 5d, the same mass loss was obtained for the first degradation step at 260 °C, indicating that the same amount of piperidinium groups is lost for the pristine and the KOH treated sample (26.7 wt% and 26.4 wt% weight loss, respectively). Consequently, within the accuracy of the TGA results, we can also conclude that the piperidinium group is not degraded after treating the membrane with 1 M KOH for four weeks.

We attribute the high alkaline stability to the stable *N*-methylpiperidinium cation attached to a long alkyl chain, which, in combination, results in excellent chemical stability in alkaline solutions.^16,40^ Furthermore, the absence of an ammonium cation in the benzylic position in the P4HexPipSt/O-PBI blend results in increased alkaline stability compared to other polystyrene-based AEMs with the ammonium group in the benzylic position.^63^ Thus, the identical TGA traces are a clear hint for the stability of the cationic headgroups even after exposure to KOH for four weeks. At higher temperatures, the carbon–carbon bonds of the polymer backbones are cleaved. Here, the TGA curves differ slightly (*Δ* ≈ 2 wt%), indicating minor differences in thermal stability. However, the shape of the curves is still almost identical, which hints that there are no substantial changes in the membrane composition. Summarizing the TGA analysis of the KOH-treated membranes, it was confirmed that the thermal degradation behavior does not change after treating the membrane with 1 M KOH at 85 °C for four weeks, rendering the membranes applicable in AEMWE. Moreover, for the following AEMWE tests, we focused on the blend membrane with a titrated IEC of 2.20 mmol g^−1^ due to the promising anion conductivity in combination with the confirmed alkaline stability resulting from applying a chemically stable anion-exchange material (P4HexPipSt) and a mechanically stable matrix (O-PBI). The chosen membrane is a good compromise between high anion conductivity and mechanical stability.

### AEMWE

**Fig. 6:**
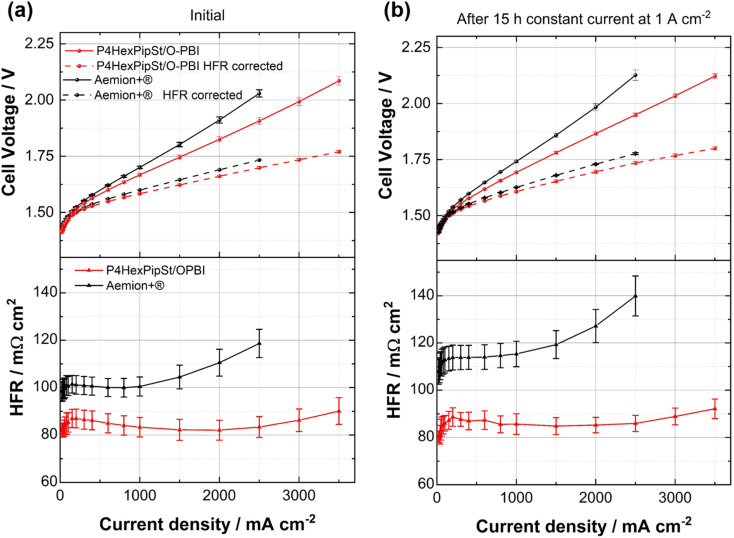
(a) Initial polarization curves and HFR values of a P4HexPipSt/O-PBI blend membrane (IEC = 2.20 mmol g^−1^, thickness = 50 μm) with HFR corrected data compared to Aemion+® AF3-HWK9-75-X 75 (thickness = 75 μm) as commercial reference. All measurements were conducted at 70 °C with 1 M KOH as feed and at ambient pressure. The error bars correspond to one standard deviation from the mean value of three independent measurements. The HFR was obtained by measuring the galvanostatic impedance at each current density and fitting it with the transmission line model. (b) Polarization curves and HFR values after applying a constant current hold at 1 A cm^−2^ for 15 h measured under the same conditions.

After confirming the alkaline stability of the prepared blend membranes, their actual performance in an anion exchange membrane water electrolyzer was tested (Fig. 6a and b).

Pt/C was used as a catalyst at the cathode, whereas the scalable non-noble first-generation NiFe-layered double hydroxide (NiFe-LDH) from Matteco was applied at the anode side. As a feed, 1 M KOH with a 30 mL min^−1^ flow rate was used, and the electrolyte was tempered to 70 °C. Due to its high conductivity, the self-synthesized P4HexPipSt/O-PBI blend membrane with a titrated IEC of 2.20 mmol g^−1^ was evaluated and compared to the commercially available Aemion+® membrane (AF3-HWK9-75-X 75). Both MEAs were tested in the same configuration, and only the membrane was changed.

**Fig. 7:**
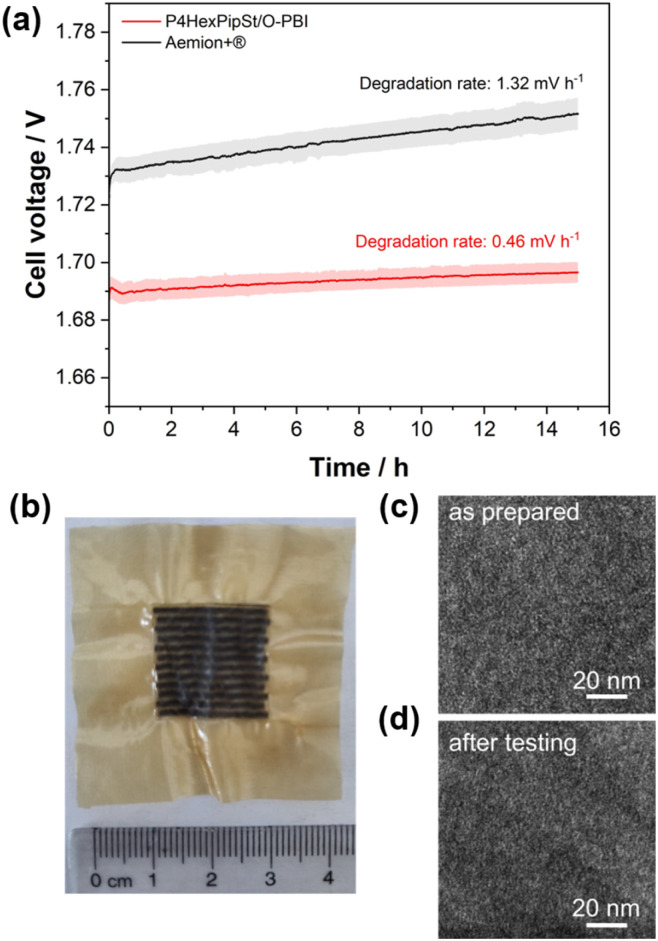
(a) Cell voltage during the constant current hold at 1 A cm^−2^ for the blend membrane and Aemion+®, whereby the shaded area represents one standard deviation from the mean value of three independent measurements. (b) Blend membrane after the constant current hold with catalyst residues on the membrane. (c) HAADF-STEM image of the membrane before testing. (d) HAADF-STEM image of the membrane after the constant current hold.

The self-synthesized P4HexPipSt/O-PBI blend membrane in a hydrated state had a thickness of 50 μm, whereas the Aemion+® membrane had a thickness of 75 μm. After a short cell break-in, the initial polarization curve was recorded with 3 minutes holding time on each point. After this holding time, a galvanostatic impedance was recorded for each current density. These impedances were fitted with an equivalent circuit based on the transmission line model introduced by Makharia *et al.*^64^ to obtain the HFR. The voltage was limited to 2.2 V to prevent degradation of the metal-containing parts of the electrolysis cell. After measuring the first polarization curve, a constant current hold at 1 A cm^−2^ for 15 h was applied to get the first information regarding the *in situ* degradation behavior of the membrane. Afterward, the polarization curve was measured again (Fig. 6a and b). The self-synthesized blend membrane achieved a high current density of 2.0 A cm^−2^ at a voltage of 1.8 V, indicating a high hydroxide conductivity. This is on par with the *ex situ* characterization, where we already confirmed a high anion conductivity (Fig. 4). When the initial polarization curve of the blend membrane is compared to the commercial reference, it is observed that the blend membrane outperforms the commercial reference in the ohmic region. This behavior is expected as the 75 μm woven reinforced Aemion+® membrane has a higher HFR than the self-synthesized 50 μm blend membrane (Fig. 6a and b). The slight difference in the HFR-corrected polarization curve could occur due to the different membrane interfaces (blend–woven reinforced) caused by the different temperature-dependent swelling behavior. By following the voltage during the 15 h constant current hold at 1 A cm^−2^, a first impression of the initial stability could be gained (Fig. 7a). Other research groups observed that the voltage increases within the first 20 h of operation due to phenyl oxidation reactions at the ionomer binder and catalyst wash-out effects.^23^ After 80 h, the voltage stabilized, and different membranes could be compared. However, in the present case, different degradation behaviors are observed for Aemion+® and the self-synthesized blend membrane. The initial voltage increase for Aemion+® is higher compared to the blend membrane. For Aemion+®, a linear voltage increase of 1.32 mV h^−1^ is observed. Moreno-Gonzáles *et al.* achieved a similar degradation rate of 0.918 mV h^−1^ with Aemion+® in a 5 cm^2^ cell within the first 150 hours of testing,^65^ experiencing the highest degradation rates at the beginning of the test. For the blend membrane, the linear voltage increase is lower compared to Aemion+® at 0.46 mV h^−1^, stabilizing to roughly 0.31 mV h^−1^ for the last 3 hours of the constant current hold. The comparatively low degradation of the self-synthesized blend membrane is also consistent with the *ex situ* stability test in 1 M KOH, where we confirmed no degradation for at least 1000 h in 1 M KOH at 85 °C (Fig. 5a). The MEA containing Aemion+® experienced a high HFR increase within the first day of testing, which must be further investigated (Fig. 6). It should be mentioned that this is a comparatively short current hold, and further long-term testing is necessary to study the degradation behavior of the membrane. The performed test is only intended to demonstrate the potential of the novel membrane in the application. Thus, for future studies, we also aim to apply longer constant current holds to investigate the membrane stability *in situ*. Nevertheless, our side-chain functionalization and blending approach resulted in highly mechanical and alkaline stable materials showing excellent *ex situ* conductivity and stability and a low HFR and *in situ* degradation rate. Thus, the performance of P4HexPipSt/O-PBI in AEMWE is consistent with the *ex situ* characterization results and the expectation that a long alkyl spacer between the polymer backbone and the cationic headgroup can enhance the conductivity and alkaline stability. Finally, the AEMWE measurements clearly show that our blend concept resulted in a high-performance AEM suitable for operating an electrolyzer at high current densities. Consequently, this study demonstrated for the first time that a blend membrane could be used in AEMWE similarly to a commercial membrane like Aemion+® and even outperforming it in the ohmic region.

Optically, the blend membrane remains unchanged after the 15 h current hold (Fig. 7b). Only residues of the catalysts are visible on the membrane surface. Moreover, the microstructure also does not change after the constant current hold, which was proven by HAADF-STEM analysis (Fig. 7c and d). Here the same homogeneous membrane composition was observed for the pristine sample (Fig. 7c and d). The EDX spectra also show the homogeneous distribution of the elements in the blend membrane (Fig. S8†). By FFT, the structure size was investigated and compared to the pristine sample, whereby, for the sample after cell testing, an average structure size of 2.8 nm was obtained. Thus, we did not observe a significant change in the structure size before cell testing (3.0 nm) compared to the sample after cell testing (2.8 nm).

The performance of the novel blend membrane in AEMWE is also comparable to other state-of-the-art materials using non-noble electrocatalysts at the anode side.^2,3,19–26,55^ For better comparison with AEMWE results from previous reports, we summarized the performance of different cells in Table 1. Compared to another blend material (PVBC-MPy/PEK-cardo, entry 1, Table 1), our blend membrane showed a significantly better cell performance since our membrane reached 2.0 A cm^−2^ at 1.8 V compared to 0.5 A cm^−2^ at 2.0 V for PVBC-MPy/PEK-cardo.^55^ Compared to ion-solvating membranes working with 24 wt% KOH, our blend approach also resulted in better cell performance (Table 1). Moreover, compared to AEMWE results from other reports utilizing commercially available membranes such as AF1-HNN8-50X (Aemion 1st gen.) or Sustainion® and ionomers like Nafion or AP1-HNN8 (entry 5 and 6, Table 1) the results obtained for our blend membrane are very promising, since also higher current densities were reached at lower voltages. However, it should be mentioned that the measurement conditions in the cited literature were different from our AEMWE characterization. Finally, we also obtained significantly better AEMWE performance than recently published polydiallylammonium interpenetrating cationic network ion-solvating membranes (entry 7, Table 1). The highest reported current densities for AEMWE were 7.7 A cm^−2^ at 2.0 V and 5.3 A cm^−2^ at 1.8 V (entries 3 and 4, Table 1). In both cases, optimized electrodes and self-designed ionomers were used to improve the cell performance, which was not the subject of the present study. However, we expect the actual cell performance to be further improved by adjusting the MEA manufacturing to the membrane properties.

**Table 1:** Comparison of different state of the art AEMWE cells with their respective cell characteristics

Study	Anode catalyst	Cathode catalyst	Ionomer	Membrane	Feed	Cell temperature	Cell voltage	Current density	Ref.
1	NiFe-LDH/NF	MoNi/NF	—	PVBC-MPy/35%PEK-cardo	1 M KOH	60 °C	2.0 V	0.5 A cm^−2^	55
2	RANEY®-type Ni	RANEY®-type Ni–Mo	—	*m*-PBI	24 wt% KOH (≈4.3 M)	80 °C	1.8 V	1.7 A cm^−2^	28
3	IrO_2_	Pt/C	PFTP-8/PFBP-14	PFTP-13	1 M KOH	80 °C	2.0 V	7.7 A cm^−2^	20
4	NiFe-nanofoam	PtRu	TMA-70	HTMA-DAPP	1 M KOH	60 °C	1.8 V	5.3 A cm^−2^	23
5	NiFe_2_O_4_	NiFeCo	Nafion	Sustainion®	1 M KOH	60 °C	2.1 V	2.0 A cm^−2^	25
6	IrO_2_	Pt/C	AP1-HNN8	AF1-HNN8-50X	1 M KOH	50 °C	1.8 V	1.0 A cm^−2^	22
7	IrO_2_	PtRu/C	TMA-70	BD3/50EVOH	1 M KOH	70 °C	2.0 V	1.6 A cm^−2^	66
8a	NiFe-LDH	Pt/C	AP1-HNN8	P4HexPipSt/O-PBI	1 M KOH	70 °C	1.8 V	2.0 A cm^−2^	This work
8b	NiFe-LDH	Pt/C	AP1-HNN8	Aemion+®	1 M KOH	70 °C	1.8 V	1.5 A cm^−2^	This work

## Conclusion

This study presents a promising approach toward high-performance anion exchange membranes using a polymer blend strategy. Novel polystyrene-based polymers bearing the cationic headgroup in a C6-spaced side chain were prepared by a functionalized monomer strategy.

The anion exchange polymer was blended with the polybenzimidazole O-PBI in the next step to get mechanically stable membranes. Interestingly, this blending approach made it possible to get mechanically stable and homogeneous membranes out of the initial water-soluble and brittle cationic styrene polymer. Consequently, this study showed that a blending approach is suitable for combining the properties of two completely different materials. The blend membranes obtained combine the benefits of the two precursor materials. Another stabilizing effect in the blend membranes is the formation of ionic crosslinks due to the deprotonation of O-PBI in highly alkaline media. By carefully adjusting the ratio of cationic polymer to O-PBI, highly conductive membranes are obtained, which are further stabilized by the *in situ* formation of ionic crosslinks. The blend membranes showed no conductivity decrease after exposure to 1 M KOH at 85 °C for six weeks. This result is on par with the TGA analysis of a blend membrane exposed to 1 M KOH at 85 °C for four weeks because the TGA curves were almost identical to the respective curve of the untreated membrane. Due to the insolubility of the blend membranes, degradation analysis of blend membranes *via* NMR spectroscopy was not possible, but NMR analysis of aged pure P4HexPipSt indicates no degradation of the piperidinium group.

Since the blend membranes were still ductile after the KOH exposure, we also can state that the O-PBI component keeps its excellent mechanical properties under the investigated conditions. Finally, the blend membranes were successfully tested in an anion exchange membrane water electrolyzer. Excellent performance exceeding Aemion+® as a commercial reference was confirmed. The electrolysis cell was run for 15 h at a constant current of 1 A cm^−2^, whereby only a slight voltage increase was measured, most likely due to effects at the electrodes rather than in the membrane. Consequently, this study showed the potential of blend membranes in alkaline electrochemical applications such as AEMWE. A novel material class with promising properties was introduced by exploiting the high IEC of the unexplored side chain functionalized cationic polystyrene and the excellent mechanical and chemical stability of O-PBI. Furthermore, the water uptake could be limited by covalent crosslinking, which will probably further stabilize the blend membranes in addition to the ionic crosslinks.

## Author contributions

Linus Hager: conceptualization, methodology, investigation, data curation, visualization, writing – original draft. Manuel Hegelheimer: methodology, investigation, data curation, writing – review & editing. Julian Stonawski: writing – review & editing. Anna Freiberg: supervision, writing – review & editing. Camilo Jaramillo-Hernández: NiFe-LDH synthesis, scale-up, and characterization. Gonzalo Abellán: NiFe-LDH supervision, writing – review & editing, funding acquisition. Andreas Hutzler: TEM analysis, writing – review & editing. Thomas Böhm: ultramicrotomy, writing – review & editing. Simon Thiele: supervision, writing – review & editing. Jochen Kerres: supervision, conceptualization, writing – review & editing, funding acquisition.

## Conflicts of interest

There are no conflicts of interest to declare.

## Supplementary Material

TA-011-D3TA02978F-s001
