# Supplementary material for: Novel side chain functionalized polystyrene/O-PBI blends with high alkaline stability for anion exchange membrane water electrolysis (AEMWE)
Source: J Mater Chem A Mater. 2023 Oct 11;11(41):22347–59. doi: 10.1039/d3ta02978f (PMC10597322; doi:10.1039/d3ta02978f)
Supplement: TA-011-D3TA02978F-s001 [file TA-011-D3TA02978F-s001.pdf]

Electronic Supplementary Material (ESI) for Journal of Materials Chemistry A.
This journal is © The Royal Society of Chemistry 2023

Supporting Information

Novel Side Chain Functionalized Polystyrene/O-PBI Blends with High Alkaline Stability for Anion Exchange Membrane Water Electrolysis (AEMWE)

Linus Hager,*[ab] Manuel Hegelheimer,[ab] Julian Stonawski,[ab] Anna T.S. Freiberg,[ab] Camilo Jaramillo-Hernández,[c] Gonzalo Abellán,[c] Andreas Hutzler,[a] Thomas Böhm,[a] Simon Thiele [ab] and Jochen Kerres*[ad]

*Corresponding authors

Materials:

The polybenzimidazole O-PBI with a number/mass average molecular weight of M_n/M_w = 170/339 kg mol^{-1} was obtained from FUMATECH BWT GmbH. 4-chlorostyrene was purchased from Manchester Organics. Magnesium, tetrahydrofuran (dry), 1,2-dibromoethane, 1,6-dibromohexane, lithium bromide, copper(I) bromide, diethyl ether, 2,2'-azobis(2-methylpropionitrile) (AIBN), dimethylformamide, dimethyl sulfoxide, acetonitrile, sodium tungstate and magnesium sulfate were purchased from Sigma Aldrich and used without further purification. 1-methylpiperidine was obtained from Thermo Fisher Scientific and used without further purification.

Monomer synthesis:

Synthesis of 4-(6-bromohexyl) styrene: Under an argon atmosphere, Mg (5.669 g, 233.2 mmol, 1.06 eq.) was added to a three-neck round bottom flask. Subsequently, 4-Chlorostyrene (30 mL, 0.75 M in THF) was added. The mixture was heated to reflux, and a few drops of 1,2-dibromoethane were added to start the reaction. While keeping the reaction mixture under reflux, the remaining 4-Chlorostyrene (263 mL, 0.75 M in THF) was added dropwise within 1 h. After stirring for a further 90 min under reflux, the reaction was allowed to cool to room temperature and stirred for another hour. After that, the Grignard solution obtained was used without further purification or analysis. LiBr (4.343 g, 50.00 mmol) and CuBr (3.586 g, 25.00 mmol) were dissolved in dry THF (50 mL) to form the active catalyst ($LiCuBr_2$). The $LiCuBr_2$ solution (27 mL) was added to a solution of 1,6-dibromohexane (220.1 g, 902.0 mmol, 4.10 eq.) in dry THF (361 mL) and cooled to 0 °C. The styryl Grignard was added slowly to the 1,6-dibromohexane solution over 2 h. Afterward, the solution was stirred at room temperature for 16 h and then quenched by the addition of NaCN (14.432 g, 294.47 mmol) and NH_4Cl (90.200 g, 1.686 mol) dissolved in ultrapure water (631 mL). The aqueous phase was extracted twice with diethyl ether (200 mL), and the combined organic phases were washed twice with ultrapure water (200 mL). After drying the combined organic phases over $MgSO_4$, diethyl ether was removed under reduced pressure. The residual 1,6-dibromohexane was removed by vacuum distillation. The crude product was purified by flash column chromatography with cyclohexane as eluent to obtain the pure product as a colorless oil—yield: 32.32 g (55 %). ^1H NMR (500 MHz, $CHCl_3$-*d*, δ): 7.34 (d, J = 8.2 Hz, 2H; Ar *H*), 7.14 (d, *J* = 8.2 Hz, 2H; Ar *H*), 6.72 (dd, *J* = 17.6, 10.9 Hz, 1H; C*H*=CH$_2$), 5.72 (d, *J* = 17.6 Hz, 1H; CH=C*H*$_2$), 5.20 (d, *J* = 10.9 Hz, 1H; CH=C*H*$_2$), 3.42 (t, *J* = 6.8 Hz, 2H; C*H*$_2$-Br), 2.62 (t, *J* = 7.7 Hz, 2H; C*H*$_2$-Ar), 1.89 (m, 2H, C*H*$_2$-CH$_2$-Br), 1.66 (m, 2H, C*H*$_2$-CH$_2$-Ar), 1.50-1.45 (m, 2H, C*H*$_2$-CH$_2$-CH$_2$-Br), 1.39-1.34 (m, 2H, C*H*$_2$-CH$_2$-CH$_2$-Ar). ^{13}C NMR (125 MHz, $CHCl_3$-*d*, δ): 142.5 (*C*$_{ar}$-CH$_2$), 136.8 (*C*H=CH$_2$), 135.3 (*C*$_{ar}$-CH=CH$_2$), 128.7 (*C*$_{ar}$-C$_{ar}$-CH$_2$), 128.7 (*C*$_{ar}$-C$_{ar}$-CH$_2$), 126.3 (*C*$_{ar}$-C$_{ar}$-CH=CH$_2$), 126.3 (*C*$_{ar}$-C$_{ar}$-CH=CH$_2$), 113.0 (*C*H$_2$=CH), 35.6 (*C*H$_2$-C$_{ar}$), 34.1 (*C*H$_2$-Br), 32.8 (*C*H$_2$-CH$_2$-Br), 31.3 (*C*H$_2$-CH$_2$-C$_{ar}$), 28.5 (*C*H$_2$-CH$_2$-CH$_2$-Br), 28.1 (*C*H$_2$-CH$_2$-CH$_2$-C$_{ar}$).

Synthesis of 1-methyl-(6-(4-vinylphenyl) hexyl) piperidin-1-ium bromide: 4-(6-bromohexyl) styrene (7.000 g, 26.197 mmol, 1.00 eq.) was dissolved in acetonitrile (24 mL). Afterward, 1-methylpiperidine (4.800 mL, 39.300 mmol, 1.15 eq.) was added, and the reaction mixture was stirred at 50 °C for 72 h, whereby the product precipitated during the reaction. The product was separated by filtration and repeatedly washed with diethyl ether to obtain the pure product as white powder—yield: 8.372 g (87 %). ^1H NMR (500 MHz, D_2O, δ): 7.42 (d, J = 8.1 Hz, 2H; Ar *H*), 7.24 (d, J = 8.1 Hz, 2H; Ar *H*), 6.73 (dd, *J* = 17.7, 10.9 Hz; 1H, C*H*=CH$_2$), 5.77 (d, *J* = 17.7, 1H, C*H*$_2$=CH), 5.23 (d, *J* = 17.7, 1H, C*H*$_2$=CH), 3.22 (t, *J* = 5.8 Hz, 4H, C*H*$_2$-N$^+$), 3.19 – 3.14 (m, 2H, C*H*$_2$-N$^+$), 2.92 (s, 3H, C*H*$_3$-N$^+$), 2.60 (t, *J* = 7.4 Hz, 2H, C*H*$_2$-Ar), 1.81-1.78 (m, 4H, C*H*$_2$), 1.63-1.59 (m, 6H, C*H*$_2$), 1.34-1.31 (m, 4H, C*H*$_2$). ^{13}C NMR (125 MHz, DMSO-*d*$_6$, δ): 142.0 (*C*$_{ar}$-CH$_2$), 136.5 (*C*H=CH$_2$), 134.7 (*C*$_{ar}$-CH=CH$_2$), 128.5 (*C*$_{ar}$-C$_{ar}$-CH$_2$), 128.5 (*C*$_{ar}$-C$_{ar}$-CH$_2$), 126.1 (*C*$_{ar}$-C$_{ar}$-CH=CH$_2$), 126.1 (*C*$_{ar}$-C$_{ar}$-CH=CH$_2$), 113.3 (*C*H$_2$=CH), 62.3 (*C*H$_2$-N$^+$), 62.3 (*C*H$_2$-N$^+$), 59.9 (*C*H$_2$-N$^+$), 47.0 (*C*H$_3$-N$^+$), 34.7 (*C*H$_2$-C$_{ar}$), 30.6 (*C*H$_2$), 28.1 (*C*H$_2$), 25.7 (*C*H$_2$), 20.9 (*C*H$_2$), 20.7 (*C*H$_2$), 19.3 (*C*H$_2$), 19.3 (*C*H$_2$).

Polymer and membrane characterization:

Nuclear Magnetic Resonance: NMR spectra were measured at room temperature with a *JEOL* JNM-ECZ-500R with a proton resonance frequency of 500 MHz. For the different NMR measurements, samples were dissolved in deuterated chloroform ($CHCl_3$-d), deuterated dimethyl sulfoxide (DMSO-d_6) or deuterium oxide (D_2O). The signal of the residual protons in the deuterated solvent was selected as the internal standard with a shift of 7.26 ppm for $CHCl_3$-d, 2.50 ppm for DMSO-d6, and 4.79 ppm for D_2O.

Gel permeation chromatography (GPC): GPC measurements were performed using a SECcurity^2 1260 from *PSS*. A *PSS* GRAM GUARD was used *as a* guard column, and three separation columns (1x *PSS* GRAM 10 µm 100 Å and 2x *PSS* GRAM 10 µm 3000 Å) were applied for sample analysis. The eluent was DMSO containing 0.1 M LiCl with a flow rate of 1.0 mL min^{-1} at 70 °C. A dual variable wavelength UV-Vis (P/N 404-2107, PSS) and a refractive index detector (P/N 404-2106, PSS) were used as detectors. The relative molecular weight was obtained by calibration with narrowly distributed poly(methyl methacrylate) standards from *PSS.*

Thermogravimetric analysis (TGA): The thermal stability of the polymers and membranes was analyzed using a TGA 8000 from *PerkinElmer* with a heating rate of 20 K min^{-1} from 30 °C to 800 °C under a synthetic air atmosphere.

Differential Scanning calorimetry (DSC): DSC curves were obtained using a *Mettler Toledo DSC 3+*. Analyses were performed under nitrogen flow (50 mL min^{-1}) at a heating rate of 10 K min^{-1}. The method consisted of three measurement steps with stationary phases of 5 min in between: (1) heating from -50 °C until 250 °C; (2) cooling down to -50 °C and (3) repeating step 1. The glass transition temperature (T_g) was calculated according to the ISO standard method on the last heating curve.

Dynamic mechanical analysis (DMA): Tensile tests were conducted in a DMA 1 from *Mettler Toledo.* Three samples of 6x25 mm in Cl^- form for each tested membrane were prepared. The membranes were measured at 25 °C and 70 °C under dry conditions and immersed in a water bath at the same temperatures.

Electrochemical Impedance Spectroscopy (EIS): A Zennium X from Zahner was used for electrochemical impedance spectroscopy. The potentiostat was connected to an external cell having two gold electrodes with an area of 0.25 cm^2. A 1.5 x 1.5 cm membrane sample was immersed in 1 M aqueous NaCl solution at 85 °C for 24 h. After cooling to room temperature, the membrane was sandwiched by two pieces of AEMION® in a stack, and the membrane sandwich was placed flat between the two gold electrodes. Before and after the measurement, the reference membranes were measured without the sample. Single drops of 1 M NaCl solution were applied as electrolytes between every electrode or membrane interphase. Three pieces of a membrane sample were measured twice each. The resulting resistance of the reference membranes was subtracted from the stack resistance to obtain the sample resistance. The conductivity was calculated according to equation (1) whereby *d* represents the membrane thickness, *R* the measured resistance, and *A* the electrode area:

$$\sigma = \frac{d}{R \cdot A} \tag{1}$$

Hydroxide Conductivity: Membrane pieces with a thickness (d) of about 44 µm and a width (w) of about 0.5 cm were immersed in degassed 1 M KOH for 24 h to convert the membranes into their mixed hydroxide form, afterwards the membranes were washed three times with degassed DI water to remove excess KOH. The membranes were loaded into an MTS 740 (Scribner Associates) four-point probe conductivity cell. The humidity was controlled at 95 % RH, and the flow of nitrogen was at 500 sccm min^{-1}. For temperature-dependent measurements, the cell was first equilibrated at 30 °C for 1 h, and then the resistance of the membrane was measured. Then the temperature was raised by 10 °C, and the cell was equilibrated at the respective temperature for 1 h. After equilibration, the resistance was measured again. All measurements were repeated three times. The conductivity was calculated with equation (2), whereby L is the distance between the two sensing electrodes (l = 0.425 cm), R is the measured resistance, w is the width, and d is the thickness of the membrane:

$$\sigma = \frac{L}{R \cdot w \cdot d} \tag{2}$$

For measuring the hydroxide conductivity of the aged samples, the procedure was the same as for the measurement at 30 °C.

Mohr's Titration and calculation of theoretical IECs: A membrane in Cl^- form was immersed in 1 M aqueous $NaNO_3$ solution for 48 h at room temperature. Afterward, the membrane was withdrawn, and sulfuric acid (250 µL, 2 mol L^{-1}) was added to the remaining solution. The solution was titrated at room temperature with 0.01 M aqueous $AgNO_3$ solution in an OMNIS Titrator with an OMNIS Dosing Module from Metrohm. The IEC was calculated by the following equation, with V_{AgNO3} as the consumed volume of $AgNO_3$, c_{AgNO3} the concentration, and $m_{dry, OH-}$ as the dry mass of the membrane in hydroxide form:

$$\mathrm{IEC}_{titrated} = \frac{V_{\mathrm{AgNO_3}} \cdot c_{\mathrm{AgNO_3}}}{m_{\mathrm{dry,OH^-}}} \qquad (3)$$

The theoretical IEC of a blend membrane is given by the weight ratio of the positively charged anion exchange polymer m(P4HexPipSt) and O-PBI m(O-PBI). The maximum theoretical IEC is determined by the molecular weight of one repeating unit of P4HexPipSt in its hydroxide form:

$$\mathrm{IEC}_{theo} = \frac{m(\mathrm{P4HexPipSt}, OH^-)}{M(\mathrm{P4HexPipSt, OH^-}) \cdot [(m(\mathrm{P4HexPipSt}) + m(\mathrm{OPBI})]} \qquad (4)$$

Swelling Ratio (SR) and Water Uptake (WU): A wet piece of the membrane was gently swabbed with a dry paper towel, then weighed, and its thickness, d, and length, L, were measured. After drying in a vacuum at 60 °C for 24 h, the membrane was weighed and measured again. The SR_L, SR_T, and WU were calculated with the following formulas:

$$\mathrm{SR}_L = \frac{L_{wet} - L_{dry}}{m_{dry}} \cdot 100\% \qquad (5)$$

$$\mathrm{SR}_T = \frac{d_{wet} - d_{dry}}{d_{dry}} \cdot 100\% \qquad (6)$$

$$\mathrm{WU} = \frac{m_{wet} - m_{dry}}{m_{dry}} \cdot 100\% \qquad (7)$$

Transmission electron microscopy (TEM): Before TEM analysis, the membranes were immersed in 1 M Na_2WO_4 for 72 h, followed by immersion in ultrapure water for 24 h three times each. The WO_4^{2-} staining increases the contrast since the image contrast with a high-angle annular dark field detector scales with the square of the atomic number (Rutherford scattering). The membranes were embedded in epoxy resin (Araldite 502) and cut at room temperature with a Diatome ultra 45 diamond knife with water as a floating liquid on an RMC Boeckeler PowerTome. Microstructure analysis was performed using a Talos F200i (ThermoFisher Scientific). The microscope was operated at an acceleration voltage of 80 kV to reduce beam damage. High-angle annular dark field scanning transmission electron microscopy (HAADF-STEM) was utilized to exploit the mass-thickness contrast within ultra-thin sections (60 nm) of the tungstate-stained blend-polymer material. Therefore, a beam current of 25 pA and a convergence angle of 7.3 mrad were adjusted. Energy-dispersive X-ray spectroscopy (STEM-EDXS) using a Dual Bruker XFlash 6 | 100 EDS detector was, furthermore, employed to gain information about the elemental distribution within the polymer.

Additional discussion on the monomer synthesis:

The first attempts to synthesize styrene monomers with alkyl-halide functional groups in the para position by coupling styryl Grignard reagent to alkyl dihalides go back to the studies of Hallensleben from 1973.[1] Their first attempts were unsuccessful due to the formation of doubly connected products, resulting in crosslinked and insoluble polymers. The suppression of double coupling is only possible by the usage of a catalyst.[2]
Typically, Li_2CuCl_4 or $LiCuBr_2$ are used to ensure a fast coupling reaction and, thus, to prevent double coupling.[3] Consequently, we focused on the synthesis originally published by Bertini *et al*. with $LiCuBr_2$ as the active catalyst. We obtained 4-(6-bromohexyl) styrene by coupling the Grignard reagent from 4-chlorostyrene to a significant excess of 1,6-dibromohexane in a yield of 55 %.

Kharasch and Fields described the coupling of two aryl Grignard reagents by metal halide in 1941.[4] The synthesis was repeated several times, and the side product always was observed in the ^{1}H-NMR spectrum in the same amount of around 2 mol%. Consequently, all free radical polymerization attempts utilizing 4-(6-bromohexyl) styrene failed and resulted in insoluble gels since the by-product 4,4'-divinyl-1,1'-biphenyl effectively crosslinks during the free

radical polymerization. The benzylic protons' integral (**Figure S1**, H-6) and the CH_2-Br group (**Figure S1**, H-5) match perfectly within NMR spectroscopy's accuracy. Furthermore, all relevant signals of the target compound are also present in the ^{13}C NMR spectrum (**Figure S2**). Thus, the styryl Grignard was coupled to only one equivalent 1,6-dibromohexane because of the high excess of 1,6-dibromohexane.

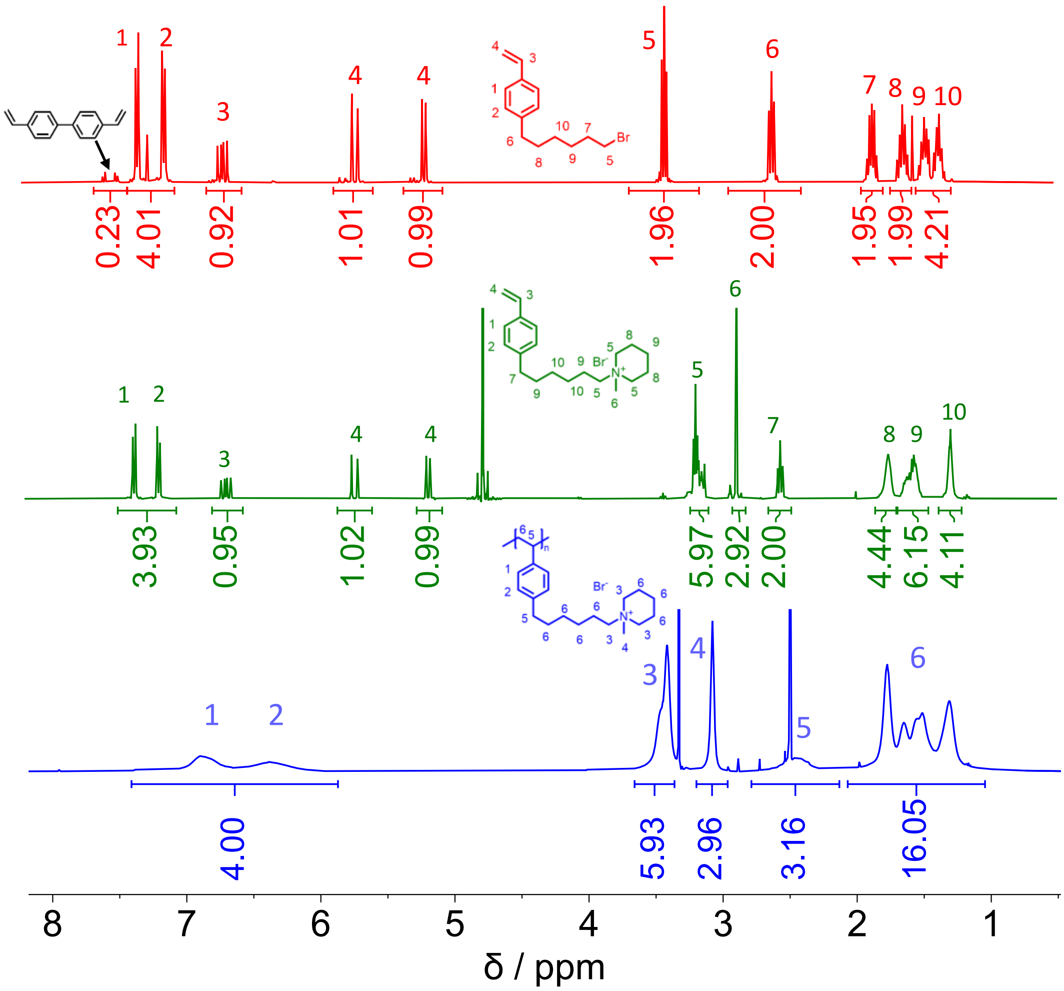

Figure S 1: ^{1}H NMR spectra of 4-(6-bromohexyl) styrene (b) 1-methyl-(6-(4-vinylphenyl) hexyl) piperidin-1-ium bromide (c) poly(1-methyl-(6-(4-vinylphenyl) hexyl) piperidin-1-ium bromide).

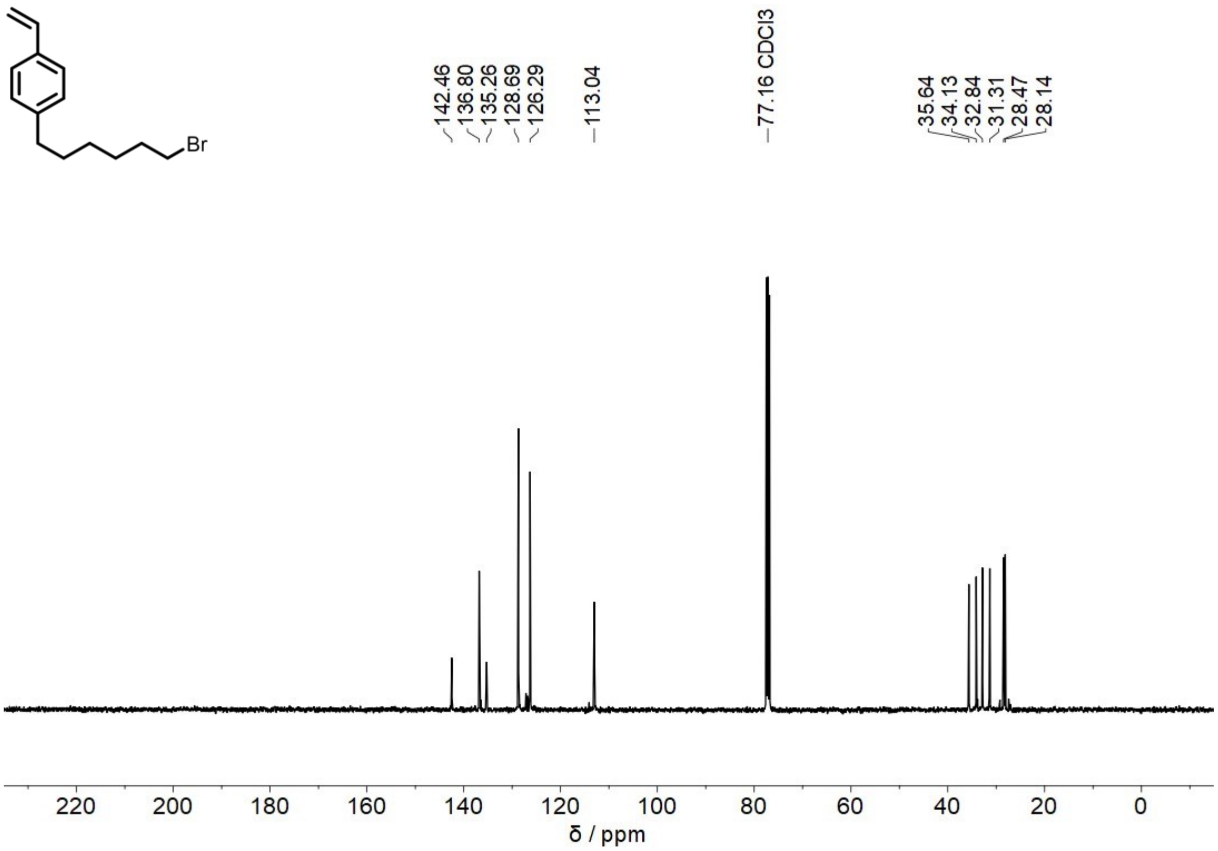

Figure S 2: ^{13}C NMR spectrum of 4-(6-bromohexyl) styrene measured in $CHCl_3$-d.

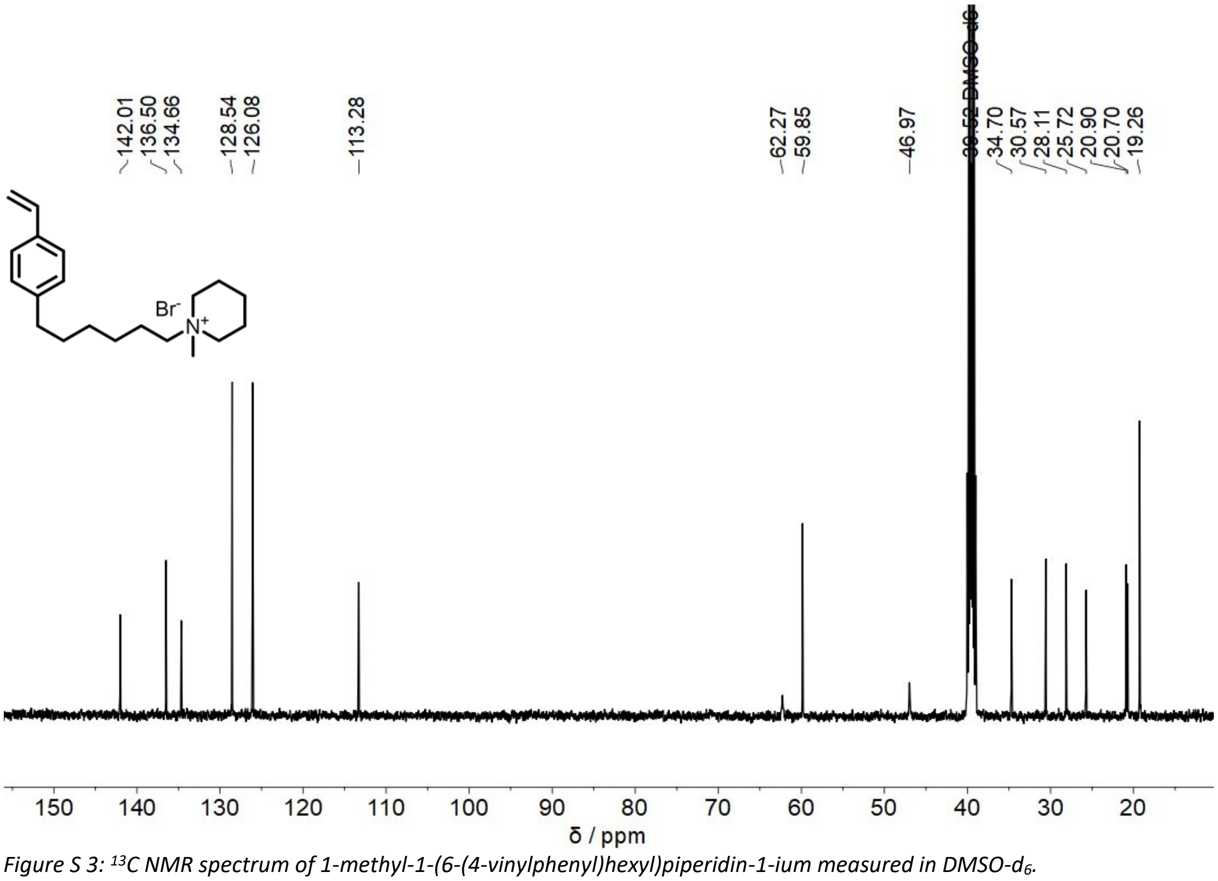

Figure S 3: ^{13}C NMR spectrum of 1-methyl-1-(6-(4-vinylphenyl)hexyl)piperidin-1-ium measured in DMSO-d_6.

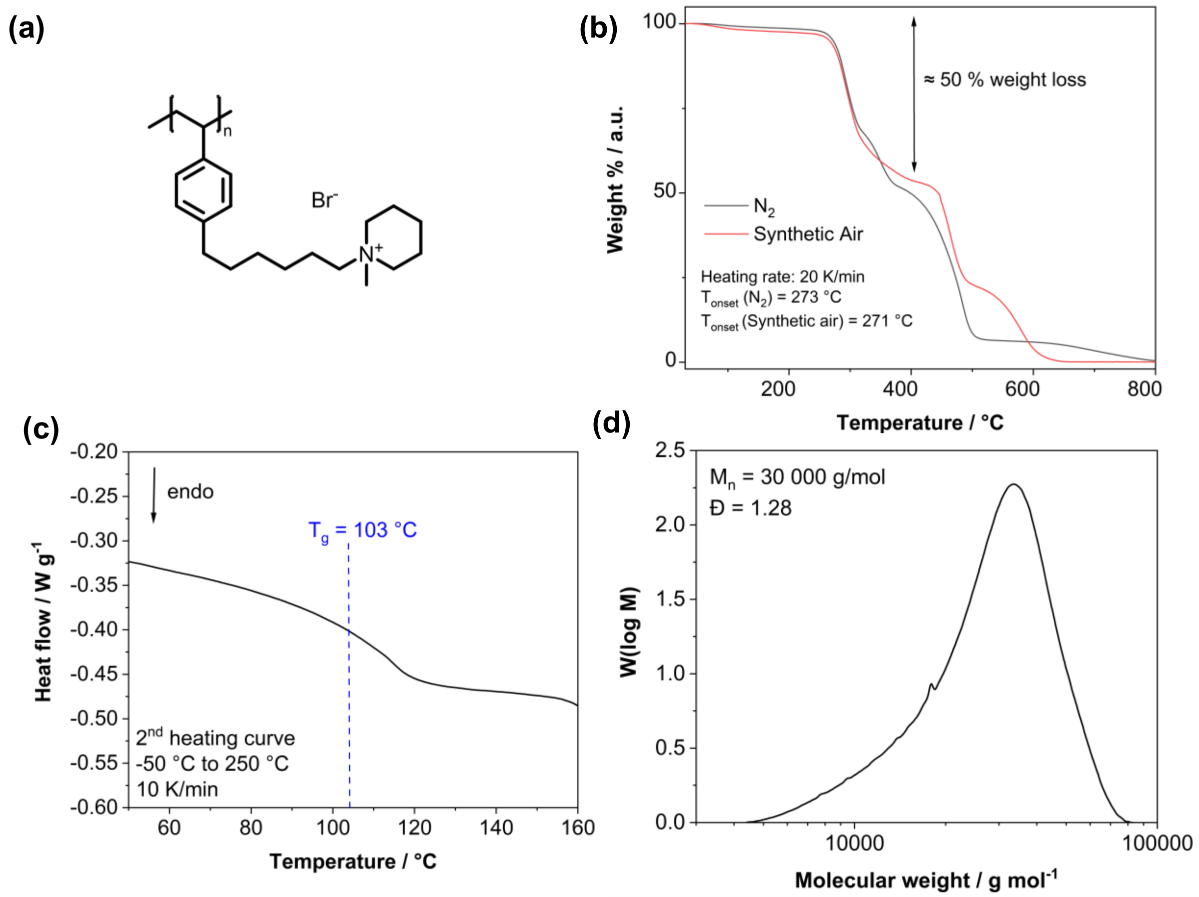

Figure S 4: (a) Structure of poly(1-methyl-(6-(4-vinylphenyl) hexyl) piperidin-1-ium bromide) (b) TGA curves of the polymer measured under nitrogen and synthetic air (c) DSC curve (d) GPC curve measured with 0.1 M LiCl in DMSO with a flow rate of 1 mL min^{-1} calibrated against narrowly distributed PMMA standards

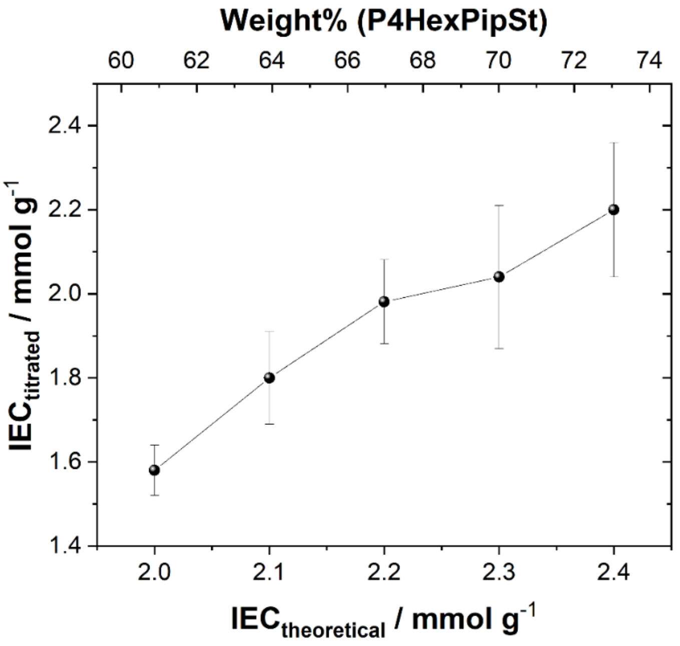

Figure S 5: Illustration of the titrated IEC depending on the theoretical IEC determined by the amount of P4HexPipSt in the polymer blend.

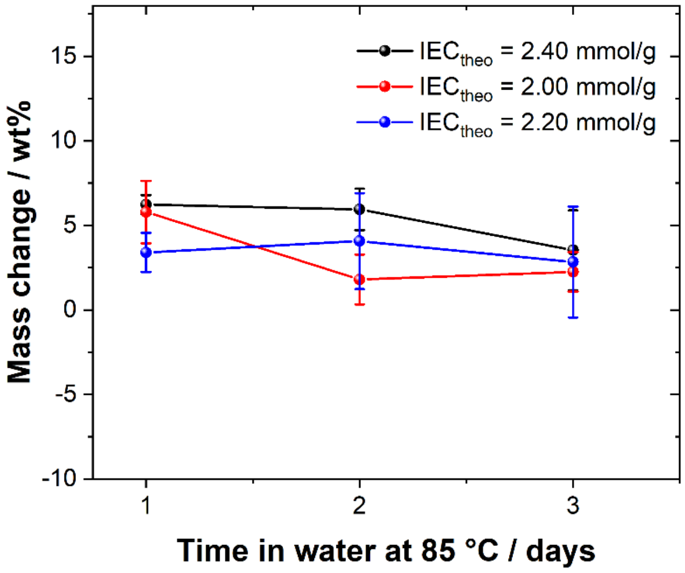

Figure S 6: Relative mass changes of blend membranes with different IECs indicating no dissolution of the water-soluble P4HexPipSt.

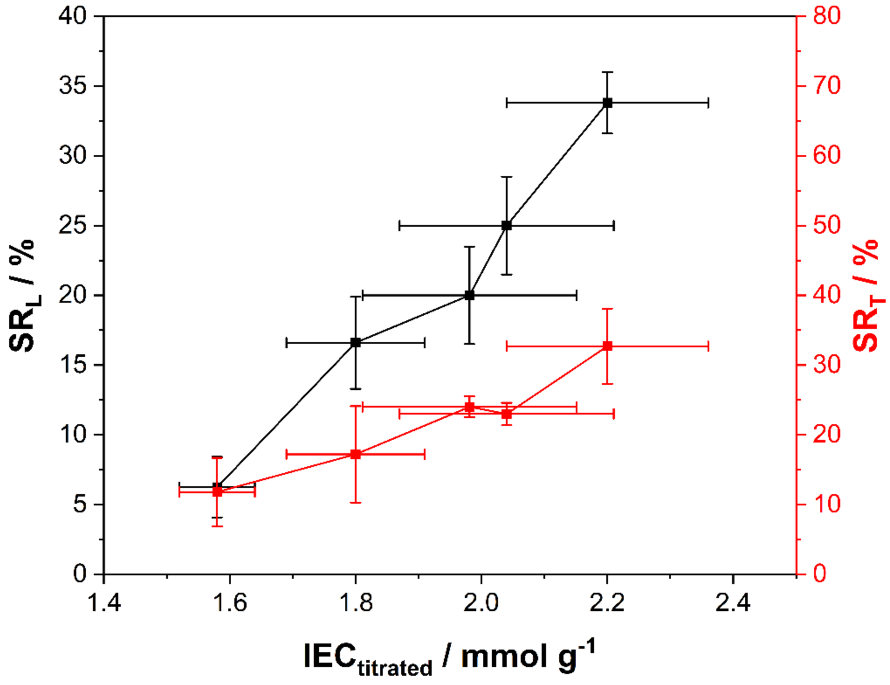

Figure S 7: Swelling ratio in longitudinal (SR_L) direction and transversal (SR_T) direction in dependency of the IEC measured at 85°C in DI water.

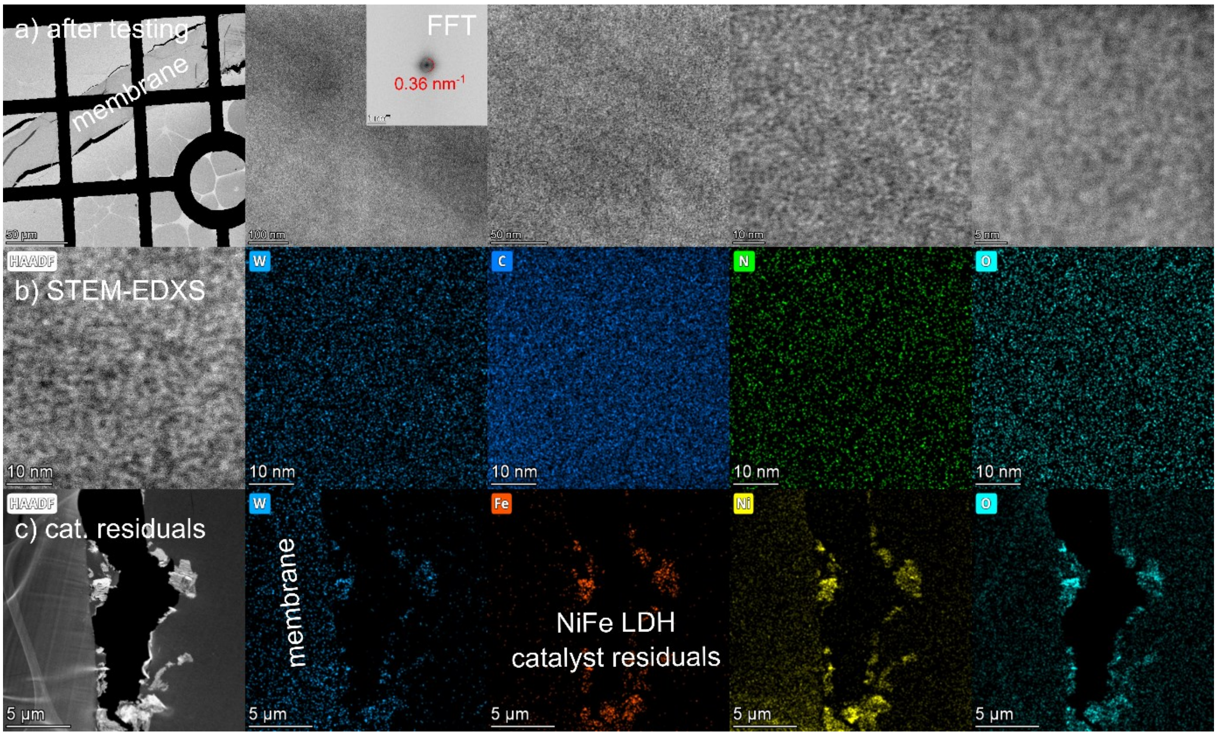

Figure S 8: HAADF-STEM images of membrane polymer after full-cell testing, b) STEM-EDX spectrum images showcasing the homogeneous distribution after testing of C, N, and O, as well as W utilized for staining, and c) spectrum images of NiFe LDH catalyst residuals. Structure size within the polymer were evaluated via the radial profiles of the fast Fourier transformed micrographs (FFT) shown in a).

Table S 1: Thicknesses of blend membranes with different weight ratios of P4HexPipSt and O-PBI.

**Entry**	**Weight% P4HexPipSt**	**IEC_{theo}**	**$IEC_{titrated}$**	**Thickness(dry)**	**Thickness(wet)***
1	61 wt%	2.00 mmol g^{-1}	1.58 mmol g^{-1}	(36.5±0.5) µm	(40.8±2.0) µm
2	67 wt%	2.20 mmol g^{-1}	1.98 mmol g^{-1}	(39.0±0.7) µm	(48.4±1.0) µm
3	73 wt%	2.40 mmol g^{-1}	2.20 mmol g^{-1}	(39.3±1.9) µm	(52.2±3.3) µm

*In DI water

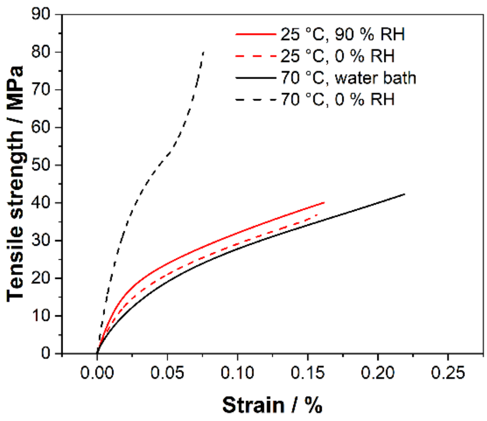

Figure S 9: Stress-strain curves of pure O-PBI under different conditions (0 % RH, 90 % RH, fully hydrated) and different temperatures (25 °C and 70 °C).

All DMA curves display a similar shape except those measured at 70 °C and 0 % RH. This probably can be attributed to the plasticizing effect of adsorbed water. In the case of 70 °C and 0 % RH, this water is expected to be removed, leading to a stiffer material. This is also reflected by the Youngs Moduli summarized in the following table:

Table S 2: DMA analysis of pure O-PBI with the respective Youngs Moduli.

Entry	RH	Temperature	Youngs Modulus
1	0 %	25°C	684 MPa
2	90 %	25°C	883 MPa
3	0 %	70°C	1674 MPa
4	Water bath	70°C	561 MPa

In contrast, we observed increased water uptake for the blend membranes accompanied by a significant plasticizing effect under humidified conditions (90 % RH, 25°C and water bath, 70°C). This is due to the water absorption of the quaternary ammonium groups. Unfortunately, we could not measure P4HexPipSt as a pure sample due to its water solubility and brittleness.

[1] M. L. Hallensleben, *Die Angewandte Makromolekulare Chemie* **1973**, *31*, 147.

[2] H. Andringa, J. Hanekamp, L. Brandsma, *Synth. Commun.* **1990**, *20*, 2349.

[3] V. Bertini, S. Alfei, M. Pocci, F. Lucchesini, N. Picci, F. Iemma, *Tetrahedron* **2004**, *60*, 11407.

[4] M. S. Kharasch, E. K. Fields, *J. Am. Chem. Soc.* **1941**, *63*, 2316.
